# Phase Behavior of Aqueous Biphasic Systems with Choline Alkanoate Ionic Liquids and Phosphate Solutions: The Influence of pH

**DOI:** 10.3390/molecules26061702

**Published:** 2021-03-18

**Authors:** Paula Berton, Hongzhe Tian, Robin D. Rogers

**Affiliations:** 1 Department of Chemistry, McGill University, 801 Sherbrooke St. West, Montreal, QC H3A 0B8, Canada; paula.berton@ucalgary.ca; 2 Chemical and Petroleum Engineering Department, University of Calgary, 2500 University Drive NW, Calgary, AB T2N 1N4, Canada; 3 Plant Protection College, Shenyang Agricultural University, 120 Dongling Road, Shenyang 110161, China; 4 525 Solutions Inc., P.O. Box 2206, Tuscaloosa, AL 35403, USA

**Keywords:** aqueous biphasic system, choline alkanoate ionic liquid, pH effect, phosphate solution, triazine-based herbicides

## Abstract

Aqueous biphasic systems (ABS) composed of the choline alkanoate ionic liquids (ILs) choline acetate [Cho][OAc], choline propanoate [Cho][Pro], choline butyrate [Cho][But], and choline hexanoate [Cho][Hex], mixed with K_3_PO_4_ solutions at pH 7.2 and 14.5, were prepared and their phase diagrams were compared. The ability to form ABS with alkaline K_3_PO_4_ solutions decreased in the order [Cho][OAc] ≈ [Cho][Pro] > [Cho][But] > [Cho][Hex], while with neutral K_3_PO_4_ solutions, [Cho][OAc] could not form an ABS, and the other three ILs performed similarly. All of the biphasic regions of the ABS decreased with the increase in pH. ^1^H-NMR data indicated anion exchange between phases in ABS at neutral pH. The ABS at neutral pH were evaluated to extract the triazine herbicides simazine, cyanazine, and atrazine, and the ABS formed by [Cho][Pro] and the pH 7.2 K_3_PO_4_ solution has shown extraction recoveries higher than 90%.

## 1. Introduction

Aqueous biphasic systems (ABS) are, as the name implies, two-phase systems mainly composed of water. The aqueous nature of the two phases, without using volatile organic solvents, suggests that these systems could be more environmentally friendly alternatives for traditional liquid–liquid extraction methods. ABS have been reported for extractions of organic compounds [1,2,3,4,5,6] and biological macromolecules [7,8,9,10]. Based on their components, these systems can be classified as polymer/salt, polymer/polymer, ionic liquid (IL)/polymer, IL/salt, and IL/surfactant. The ABS based on polymers generate systems with high viscosity, which can be a drawback in extraction processes. Therefore, ABS with salts and ILs are preferable choices and by their nature have more tunable properties [11].

Ionic liquids, defined as organic salts with melting points below 100 °C [12], are salts that can be designed to have tunable properties for specific applications, including sample pretreatment and analyte extraction [13]. At present, most of the ABS with salts and ILs focus on systems with imidazolium-based ILs and inorganic salts [14,15]; however, some of the imidazolium-based ILs can have negative impacts on the environment and organisms [16,17]. Therefore, it is very important to design ABS based on biocompatible, environmentally friendly ILs.

Choline-based ILs are biodegradable, present low toxicity, are readily available, and have a low cost [18,19,20,21,22]. However, due to their high affinity for water, these ILs are usually studied in combination with polyethylene glycol (PEG) or polypropylene glycol (PPG) to form ABS [23,24,25,26,27]. On the other hand, there is only a limited number of reports about choline-based ILs applied to develop ABS with inorganic or organic salts [28,29,30,31].

Many factors influence the formation of ABS by ILs and salts, such as the nature of the ions, mass fractions, pH, and temperature. Among these, when ABS are used in extraction processes, it is very important to examine the effect of pH, not only on the ABS formation, but also on the analytes themselves. For example, the pH can affect the electrostatic and hydrophobic interactions in the target proteins and thus influence their extraction recoveries by ABS [8,10,32,33,34,35,36,37,38,39]. However, the effect of pH on ABS formation with choline-based ILs and salts is scarcely studied [28], since it is relatively difficult to create ABS using choline-based ILs with salts.

It has been reported that high charge density salts, such as K_3_PO_4_ [28], can be used to overcome the high hydrophilicity of choline-based ILs and form ABS. However, the alkaline nature of the resulting ABS limits its application to the extraction of organic pollutants. It is reported that neutral phosphate buffer solution and neutral citrate buffer solution could salt out choline salicylate and benzyldimethyl(2-hydroxyethyl)ammonium chloride to form an ABS [28]. Ideally, the ABS should be formed using choline-based ILs and neutral buffer solutions for extractions.

Choline alkanoate ILs are environmentally friendly and easily biodegradable [19], have been used to induce ABS formation with polymers [23,26,40,41], and have shown good performance for extracting plant biocomposites [42,43]. In this work, we explore using choline acetate [Cho][OAc], choline propanoate [Cho][Pro], choline butyrate [Cho][But], choline hexanoate [Cho][Hex], and choline octanoate [Cho][Oct] in combination with K_3_PO_4_ solutions to form ABS at neutral (7.2) or alkaline (14.5) pH. Phase diagrams and a deep characterization using Nuclear Magnetic Resonance (NMR) spectroscopy are presented. Continuing our efforts to utilize ABS for the extraction of triazine-based herbicides [44,45], we also show the applicability of these ABS to the determination of these analytes.

## 2. Results and Discussion

Using the procedure described in the experimental section for the determination of the phase diagrams, a screening step was initially conducted to select the proper phosphate salt able to generate ABS with choline alkanoate ILs. Various aqueous phosphate solutions (Table 1) were added to the aqueous solutions of the ILs to form cloudy or clear solutions. The results indicated that the choline alkanoate ILs could not be salted out by sodium-based salts, irrespective of the phosphate speciation induced by pH changes. On the other hand, K_3_PO_4_ solutions adjusted to pH 7.2 or 14.5 salted out [Cho][Pro], [Cho][But], and [Cho][Hex], while no ABS could be formed with [Cho][Oct], irrespective of the pH or salt used. Phosphate salt precipitated in the system formed with [Cho][Oct] under neutral pH conditions, but no salting out was observed using the same IL under alkaline conditions. [Cho][OAc] could be salted out at pH 14.5, but not at pH 7.2. Out of the ILs tested in this work, only [Cho][OAc] was previously reported to form ABS in combination with K_3_PO_4_ under alkaline conditions (pH > 12) [28], resulting in a phase diagram and binodal curves similar to the ones reported in this work.

The binodal curves for the seven possible ABS were then constructed by cloud point titration of 80 wt% aqueous solutions of [Cho][OAc], [Cho][Pro], [Cho][But], and [Cho][Hex] mixed with aqueous K_3_PO_4_ solutions at pH 14.5 or 7.2. The alkaline phosphate solution was prepared by directly adding the appropriate amount of K_3_PO_4_ into deionized (DI) water, while the neutral phosphate solution was formed by mixing the appropriate amount of K_3_PO_4_ and H_3_PO_4_ with DI water. The phosphate solution was then dripped into the IL solution to form cloudy solutions, and water was then added to clarify the solutions. The procedure was repeated and the mass fractions of IL and K_3_PO_4_ were recorded to establish the phase diagrams.

**Figure 1:**
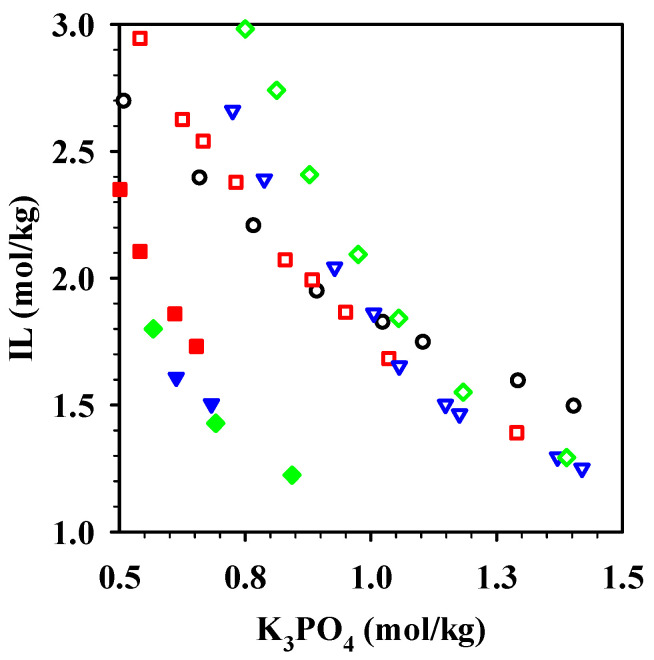
Phase diagrams of the ABS formed by [Cho][OAc] (●), [Cho][Pro] (■), [Cho][But] (▼), and [Cho][Hex] (◆) with K_3_PO_4_ at pH 7.2 (filled symbols) and pH 14.5 (open symbols). The full phase diagram is included in Supplementary Materials Figure S2.

The binodal curves at room temperature are presented in Figure 1 and the binodal data are provided in the Supplementary Materials (SM). The phase forming ability with the K_3_PO_4_ pH 14.5 solutions decreased (i.e., it took higher concentrations of the inorganic salt to form an ABS) in the order [Cho][OAc] ≈ [Cho][Pro] > [Cho][But] > [Cho][Hex]. It was previously reported that an increase in the imidazolium cation alkyl chain length leads to an increase in the IL’s hydrophobic nature and therefore to a poorer affinity for water and a greater ability of the IL to form an ABS [46]. Our observations indicate that the larger the alkyl chain in the anion, the harder it was to form an ABS at basic pH, leading to the point where no ABS was formed with [Cho][Oct]. When K_3_PO_4_ pH 7.2 solutions were used, [Cho][Pro], [Cho][But], and [Cho][Hex] were more easily salted out and all exhibited essentially the same ability to form two phases. If the phase diagram is represented using the phosphate anion (instead of K_3_PO_4_) as the horizontal axis, the phase-forming ability of the selected ionic liquids follows the same pattern, although the effect of the pH is weaker (Figure S3 in the Supplementary Materials). No differences in ABS forming ability were observed for [Cho][Pro] with respect to pH, but the ILs [Cho][But] and [Cho][Hex] were salted out more easily at neutral pH.

It has typically been reported that salt anions have the greatest effect on a salt’s ability to salt out or be salted out [47,48], and the results here suggest that some protonation of the IL anions occurs at the higher pH, since the ILs would be considered the kosmotropic (salted out) salts. Prior reports focusing on the protonation of the kosmotropic (salting out) salts (citrate buffer solutions, pH 5–8) indicate that it is easier to salt out imidazolium- or quaternary ammonium-based ILs by increasing pH of the aqueous solution due to the degree of protonation of the citrate ions at different pH [49,50]. The previous literature also indicates that ABS form more easily under alkaline conditions rather than acidic or neutral conditions [51,52]. At alkali conditions, we observe that the PO_4_^3−^ anion is able to salt out all the [Cho]-based ILs, except for [Cho][Oct]. However, larger concentrations of the PO_4_^3−^ anion are needed to form ABS (i.e., a *decrease* in salting out ability) at higher pH.

This discrepancy might be due to the higher complexity of our systems, with protic salts salting out other protic salts. The speciation of the phosphate salt changes from a H_2_PO_4_^−^/HPO_4_^2−^ system at pH 7.2 to PO_4_^3−^ at pH 14.5 [53]. In addition, the hydroxide group present in the choline cation is protonated at pH 7.2 but deprotonated (i.e., presents a negative charge) at pH 14.5 (pKa 13.9) [54], while the anions of the ILs are weak organic carboxylic acids that, at both neutral and alkaline pH, are in their anionic forms (pKa values: 4.75–4.90) [55].

**Figure 2:**
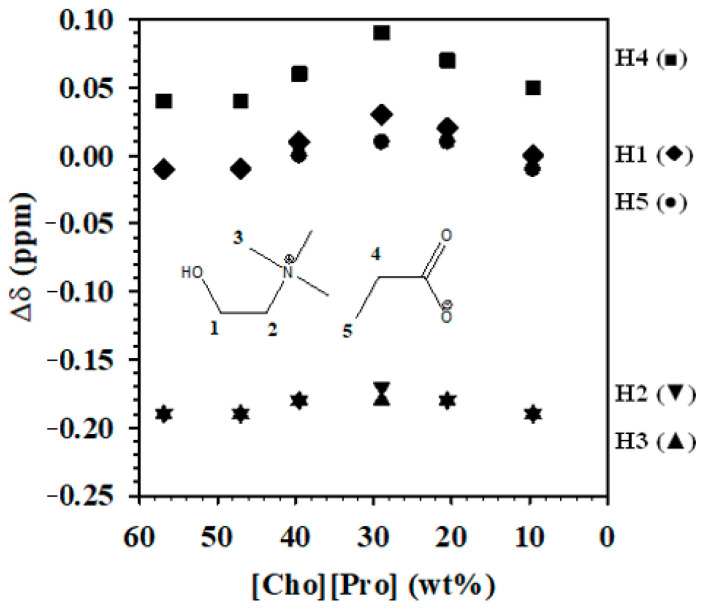
Chemical shift deviation of the protons (H1 (◆), H2 (▼), H3 (▲), H4 (■), H5 (●) following the numeration indicated in the IL formula overlapped in the figure) in aqueous solutions of [Cho][Pro] compared to pure [Cho][Pro]. Note that the chemical shifts of H2 and H3 overlap.

To further study the effect of solution pH on the speciation of IL anions, we conducted ^1^H-NMR studies of [Cho][Pro], its aqueous solutions, and the separated phases of ABS formed with K_3_PO_4_ solutions adjusted to pH 7.2 and 14.5. First, [Cho][Pro] was diluted with water to make 10–57 wt% [Cho][Pro] aqueous solutions and the NMR spectra of the resulting solutions were taken using an external standard. The chemical shift deviations (Δδ, see Experimental section, Equation (2)) were calculated as the change in δ compared to the pure IL [Cho][Pro] (Figure 2).

Water has the largest influence on the hydrogen atoms in the cation residing on C atoms directly bonded to the N (H3, H2). At 40 wt% water (60 wt% IL, ~7.5 H_2_O:IL mol ratio), these atoms experience a negative deviation of approximately −0.2, while the H4 hydrogen atoms in the anion on the C bonded to the carboxylate group exhibit the largest positive deviation of approximately 0.05. The H atoms on C atoms further away from the charge bearing groups are much less affected with essentially no or very little change in chemical shift. Interestingly, as the water concentration increases, all of the chemical shift deviations become more positive until 70 wt% (i.e., 30 wt% IL, ~64.5 H_2_O:IL mol ratio), where they turn more negative, eventually ending up nearly the same as they were with only 40% water.

**Figure 3:**
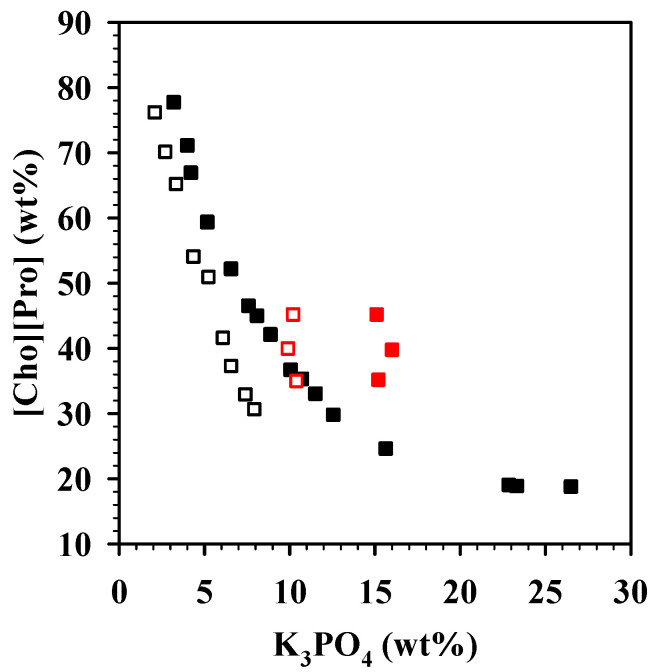
Binodal curves for [Cho][Pro]/K_3_PO_4_ ABS (shown in wt%; compare with Figure 1) at pH 7.2 (open symbols) and 14.5 (filled symbols) with the overall compositions used for the NMR study (red symbols). Tie-line data is included in Supplementary Materials Figure S4.

NMR studies were also conducted on the separated phases of the [Cho][Pro]/K_3_PO_4_ (pH 7.2 and 14.5) ABS. Three mixtures in the biphasic regions of the ABS formed under different pH were prepared by mixing K_3_PO_4_ solution and aqueous [Cho][Pro] solution, in which the mass fraction of K_3_PO_4_ was approximately 10.0 wt% at pH 7.2 or approximately 15.0 wt% at pH 14.5, while [Cho][Pro] concentrations were approximately 35, 40, or 45 wt%, respectively. The selected overall compositions are marked in red (Figure 3), overlaid on the binodal curves for these two ABS (tie-line data included in Supplementary Materials Figure S4). After phase separation, the pH of each phase (the top, IL-rich phase and the bottom, K_3_PO_4_-rich phase) was measured (Table S3 in the Supplementary Materials): When the neutral pH solution was used to generate the ABS, the resulting phases were weakly alkaline (pH 8.08–8.47), with a slightly higher pH on the top phase (8.35–8.47) than on the bottom phase (8.08–8.20), possibly indicating ion exchange among the phases. On the other hand, the two phases of the ABS formed by alkaline K_3_PO_4_ solution remained strongly alkaline (pH 13.50–14.42), with a slightly lower pH on the top phase (13.50–13.81) than on the bottom phase (14.20–14.42). In addition, the NMR spectra of the top and bottom phases of the ABS were measured (shown in the Supplementary Materials).

The NMR data of the phases formed from the [Cho][Pro]/K_3_PO_4_ ABS at different pH values were first used to follow the fate of the IL’s components. The ratio of the integral areas of the protons of the IL’s component ions was used to determine the [Cho]^+^:[Pro]^−^ ratio in the two phases (Table S4, Supplementary Materials). The cation:anion ratio remained approximately 1:1 in all of the top phases (IL-rich phases) of the ABS evaluated, irrespective of the pH of the solution and the IL concentration. The 1:1 cation:anion composition was also observed in the bottom phases of the systems at pH 14.5, while in the bottom phases (K_3_PO_4_-rich phase) of the systems at pH 7.2, the concentration of [Pro]^−^ with respect to [Cho]^+^ decreased dramatically, indicating some ion exchange between the phases at neutral pH.

**Figure 4:**
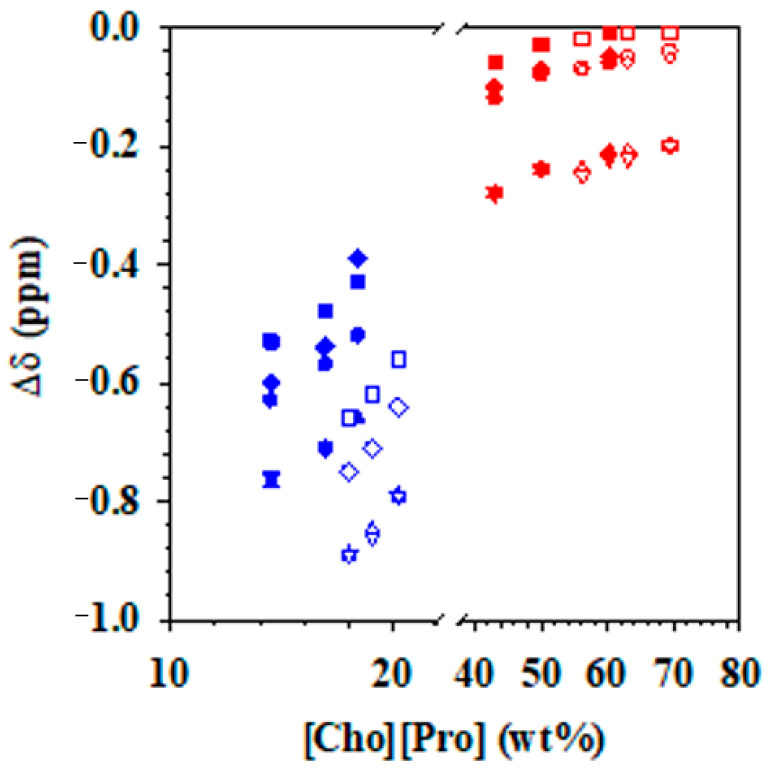
Chemical shift deviations of the protons in the top, [Cho][Pro]-rich phase (red) and the bottom, K_3_PO_4_-rich phase (blue) of the ABS with [Cho][Pro] and K_3_PO_4_ at pH 7.2 (filled symbols) and pH 14.5 (open symbols): H1 (◆), H2(▼), H3(▲), H4 (■), H5(●), following the numeration indicated in Figure 3.

The NMR data were also used to calculate the chemical shift deviations of the protons of [Cho][Pro] in the top and bottom phases of the [Cho][Pro]/K_3_PO_4_ ABS at pH 7.2 and 14.5 (Figure 4). The chemical shift deviation of all protons in the two phases was negative, which is different from the positive deviation of H5 and H1 in [Cho][Pro] aqueous solution, indicating the strong influence of the phosphate anions in the system.

In the top phases of the two ABS (Figure 4, red symbols), a downfield chemical shift deviation (negative Δδ values in comparison to pure IL) was observed for all of the groups of protons (H1 to H5) at low IL concentrations. This negative deviation decreased with the increase in IL concentration, and reached a plateau at 60 wt% IL. Comparing the deviations of the protons of the ILs in the ABS formed with alkaline solutions (Figure 4, red, open symbols) to those at neutral pH (Figure 4, red, filled symbols) indicates that the main differences are based on IL concentration rather than other effects due to the pH. The highest deviation was observed for proton H3 and H2; both protons are located in the cation. These two protons seem to interact with the water molecules at low IL concentrations, interactions that seem to decrease at higher concentrations of IL, possibly due to the formation of oligomeric ions, which is a characteristic of protic ILs.

An analysis of the protons in the bottom phases of the two [Cho][Pro]/K_3_PO_4_ ABS (Figure 4, blue symbols) indicates a downfield chemical shift deviation, i.e., the same behavior observed in the top phase but with more negative Δδ values. As described for the top phases, the shifts decrease with the increase of IL concentration, and this decrease is linear except for the H1 protons in the pH 7.2 ABS. Also, as previously described for the protons in the top phase, the H2 and H3 protons suffer the highest negative shift. Different from what was observed in the top phases, pH has a greater influence on the proton deviation, with much larger negative deviations in the system at pH 14.5 (Figure 4, blue, open symbols) than those observed in the protons of the system at pH 7.2 (Figure 4, blue, filled symbols). This difference is possibly due to the favorable interaction between the ions of the IL and water in the ABS at high pH, where the hydroxide of the choline alkyl chain is also in its negative form, forming strong ionic hydrogen-bonded complexes with water.

The NMR data of the phases formed from the [Cho][But]/K_3_PO_4_ ABS at different pH values were also collected and used to follow the fate of the IL’s components. Similar to what was observed for the [Cho][Pro]/K_3_PO_4_ ABS systems, the [Cho]^+^:[But]^−^ ratio remained approximately 1:1 in all the top phases of the ABS evaluated, but in the bottom phases of the systems, the concentration of [But]^−^ with respect to [Cho]^+^ decreased dramatically, indicating ion exchange between the phases (Table S4, Supplementary Materials). Different from what was observed with the [Pro]-based ABS, the [But]-based ABS showed a significant decrease in anions in the bottom phase, irrespective of the pH of the solution and the IL concentration.

**Figure 5:**
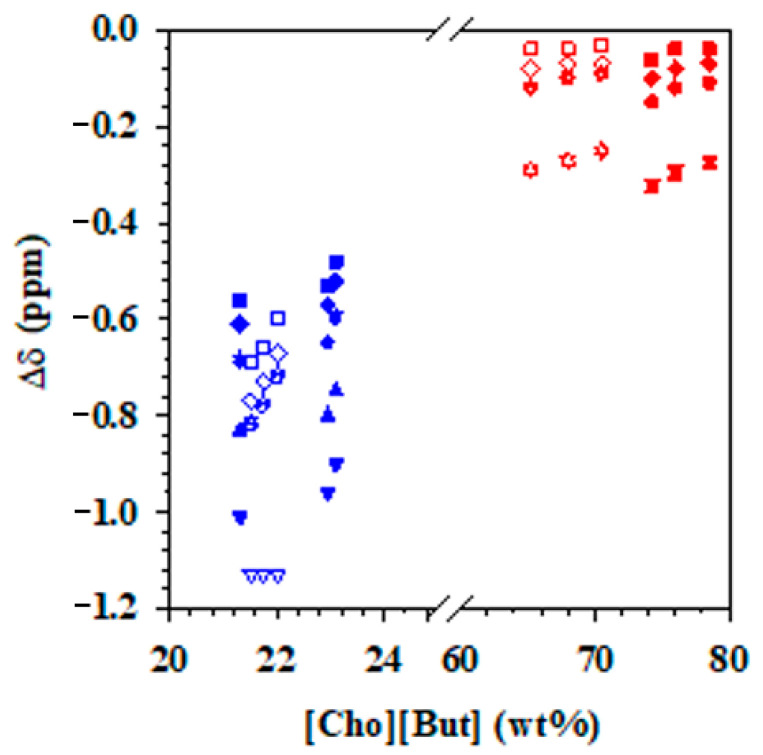
Chemical shift deviations of the protons in the top, [Cho][But]-rich phase (red) and the bottom, K_3_PO_4_-rich phase (blue) of the ABS with [Cho][But] and K_3_PO_4_ at pH 7.2 (filled symbols) and pH 14.5 (open symbols): H1 (◆), H2 (▼), H3 (▲), H4 (■), H5 (●), H6 (★), following the numeration indicated in Table 3.

The NMR data were also used to calculate the chemical shift deviations of the protons of [Cho][But] in the top and bottom phases of the [Cho][But]/K_3_PO_4_ ABS at pH 7.2 and 14.5 (Figure 5). A downfield chemical shift deviation (negative Δδ values in comparison to pure IL) was observed for all protons (H1 to H5) in the two phases, with the largest deviations observed for proton H3 and H2, as observed in the [Pro]-based ABS. In the top phases of the two ABS (Figure 5, red symbols), this negative deviation slightly decreased with the increase in IL concentration. The deviations were slightly larger for the protons of the ILs in ABS formed with neutral pH (Figure 5, red filled symbols) in comparison to those observed in alkaline solutions (Figure 5, red open symbols). The protons in the bottom phases of the [Cho][But]/K_3_PO_4_ ABS (Figure 5, blue symbols) also showed more negative Δδ values than those in top phases. Also, as previously described for the protons in the top phase, the H2 and H3 protons suffer the highest negative shift. Different from what was observed in the top phases, pH did not have an influence on the proton deviation; it seemed to be more influenced by the IL concentration than by the pH of the solution (pH 14.5, Figure 5, blue open symbols, or pH 7.2, Figure 5, blue filled symbols).

The proton behaviors in the upper and lower phases of the [Cho][Hex]/K_3_PO_4_ ABS were not analyzed since the IL components were not detected in the bottom phases (Figure S10, Supplementary Materials).

### Extraction of Triazine Herbicides Using Choline Alkanoate/K_3_PO_4_ (pH 7.5) ABS

Triazine-based herbicides such as simazine, cyanazine, and atrazine are reported to be unstable in strong acid or alkaline solutions [56]. Hence, we examined ABS comprised of [Cho][Pro], [Cho][But], and [Cho][Hex] with neutral K_3_PO_4_ solution to extract these herbicides while avoiding their degradation due to pH.

In addition to the stability of the analytes and the extraction efficiency, the viscosity of the system was another parameter to consider, since it could interfere with the mass transfer of the analytes. Hence, the viscosity of the aqueous solutions with different IL concentrations was determined (Table S5, Supplementary Materials). Although, as expected, the viscosity of the solutions increased with increasing concentrations of the IL, the viscosity values were still relatively low, even at relatively high concentrations (e.g., at over 50% IL, viscosities were only 10−12 cP). Given these results, mass transfer of the analytes during extraction should not be significantly influenced by viscosity, and fast phase equilibrium is expected, which is beneficial for the partition of the analytes between the two phases.

**Figure 6:**
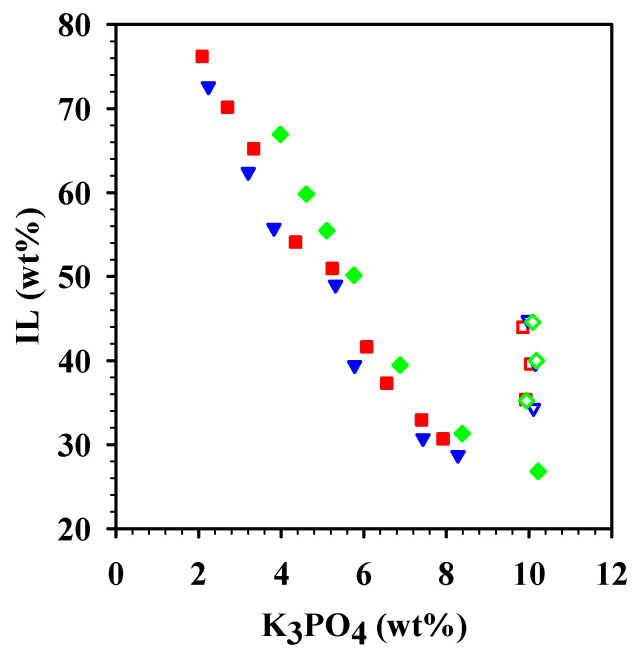
Binodal curves for ABS made with [Cho][Pro] (■), [Cho][But] (▼), and [Cho][Hex] (◆) and K_3_PO_4_ at pH 7.5 (filled symbols) with the overall compositions used for the herbicides extraction (open symbols).

The ABS were prepared with 10 wt% K_3_PO_4_ (pH 7.5) and variable amounts (35–45 wt%) of [Cho][Pro], [Cho][But], and [Cho][Hex] spiked with 4.0 µg/g of the herbicide mixture (Figure 6). After equilibration, the two phases were separated, and the herbicides were quantified using High Performance Liquid Chromatography (HPLC). In all cases, the concentrations of the herbicides that remained in the lower, K_3_PO_4_-rich phases of the ABS after extraction were below the detection limit of the HPLC, indicating high partition coefficients to the upper, IL-rich phases. Therefore, the recoveries of the herbicides extracted by the different ABS were calculated by measuring the concentration of the herbicides in the IL-rich phase (extracting phase) to evaluate the extraction efficiency of the ABS (Table 2).

The recoveries of the analytes were higher than 60% in all cases, and in most systems, the recoveries increased with increasing concentration of the ILs. Interestingly, the anion has a big effect on the extractions, and the highest recoveries were achieved in the ABS formed by [Cho][Pro] and neutral phosphate solution (recoveries higher than 85%). Also, the analytes extracted by the ABS with [Cho][Pro] and neutral phosphate solution were better separated by HPLC without significant interference compared to the ABS formed by [Cho][But] or [Cho][Hex] and neutral phosphate solutions (chromatograms of the herbicides are shown in the Supplementary Materials). Therefore, the ABS formed by [Cho][Pro] and neutral phosphate solution is the preferred system for the extraction of these analytes.

## 3. Materials and Methods

### 3.1. Materials and Chemicals

The three triazine-based herbicides simazine, cyanazine, and atrazine (≥99.0% purity), choline hydroxide solution (46% in water), glacial acetic acid (≥99.7% purity), propionic acid (≥99.5% purity), butyric acid (≥99.0% purity), hexanoic acid (≥99.0% purity), octanoic acid (≥98.0% purity), phosphoric acid (≥85.0% purity), potassium phosphate tribasic (≥98.0% purity), sodium phosphate monobasic (≥99.0% purity), sodium phosphate dibasic (≥99.0% purity), acetonitrile (HPLC grade), and methanol (HPLC grade) were purchased from Sigma-Aldrich (Oakville, ON, Canada). All chemicals and reagents used were of analytical grade or higher and were used without further purification. The deionized (DI) water used in these experiments was obtained from a Millipore purified water system (Milli-Q Academic, MilliporeSigma, Oakville, ON, Canada).

Salt aqueous solutions were prepared at the desired pH (7.2 and 14.5) by mixing appropriate amounts of potassium phosphate tribasic and phosphoric acid in DI water. Neutral phosphate solution (27.52 wt%) was prepared with 9.9590 g K_3_PO_4_ and 3.00 g H_3_PO_4_ slowly added into 23.2292 g water, stirred until completely dissolved and cooled to room temperature. Alkaline phosphate solution (48.78 wt%) was formed with 24.001 g K_3_PO_4_ added into 25.2015 g water and stirred to form a transparent solution. A similar procedure was followed to prepare the aqueous solutions of Na_2_HPO_4_ (pH 9.0), NaH_2_PO_4_ (pH 3.3), NaH_2_PO_4_/Na_2_HPO_4_ (pH 5.5), and NaH_2_PO_4_/Na_2_HPO_4_ (pH 7.0). In all cases, the pH of the solutions was measured with a pH meter (Accumet XL 600, Fischer Scientific, Ottawa, ON, Canada).

Stock standard solutions of individual herbicides (1.0 mg/mL) were prepared in methanol. An intermediate mixture of the three herbicides (100 μg/mL in methanol) was prepared by mixing the appropriate amount of the individual stock solutions, which was stored at 4 °C. Standard solutions with lower concentrations (10.0, 5.0, 2.0, 1.0, 0.5, and 0.2 μg/mL) were prepared daily in methanol by serial dilution and used for the calibration curve. A solution of 4.0 μg/mL of the three herbicides was used to study the recoveries and extraction efficiency of the ABS system.

### 3.2. Synthesis and Characterization of ILs

Choline alkanoate ILs were synthesized via neutralization of the base with the appropriate organic acids, following reported methods [42,43,57]. Briefly, 0.1 mol organic acid (glacial acetic acid, propionic acid, butyric acid, hexanoic acid, or octanoic acid) was added dropwise into an aqueous solution of choline hydroxide (0.1 mol). The mixture was stirred continuously using a magnetic stirrer (RZR 2051, Heidolph, Schwabach, Germany) for 12 h at room temperature. The obtained ILs were dried for 6 h under vacuum using a rotary evaporator (R-210, Büchi, Flawil, Switzerland), followed by freeze drying (Freezone 2.5, Labconco, Kansas City, MO, USA) for approximately 4 days.

To confirm the identity and purity of the synthesized ILs, 500 MHz NMR spectroscopy (AVIIIHD 500, Bruker, Fällanden, Switzerland) and infrared spectroscopy (Attenuated total reflection (ATR) Fourier transform infrared (FTIR) spectrometer, Alpha, Bruker, Billerica, MA, USA) were used (spectra are included in the Supplementary Materials). Viscosity was measured using a VISCOlab 3000 viscometer (Cambridge Viscosity, Inc., Boston, MA, USA) at 25 °C. The water content of the choline ILs was measured using a Karl Fisher Titrator (C20, Mettler Toledo, Greifensee, Switzerland). The chemical properties of the synthesized ILs are shown in Table 3.

### 3.3. Phase Diagrams

#### 3.3.1. Determination of the Phase Diagrams

The pure ILs (25.00 g) and 5.00 g water were added into a 100 mL beaker and mixed for 2 min, forming an approximately 80 wt% of the IL solutions. Then, 0.550 g of the 80 wt% IL in water was placed in a test tube and an aqueous solution with a known concentration of phosphate was added dropwise until the mixture became cloudy. A known mass of DI water was then added to make the mixture clear again. This procedure was repeated until enough data was obtained to develop the binodal curve of the ABS. The mass fraction of the phase components was determined by weight quantification of all the components added to the tube within an uncertainty of 0.001 g. The water content of the ILs was considered for the calculation of the compositions of the ABS mixtures. The experimental binodal data is provided in the Supplementary Materials.

#### 3.3.2. Determination of the Tie-Lines

To determine the tie-lines (TLs), mixtures in the biphasic region were prepared in 10 mL glass vials, vigorously stirred, and allowed to achieve equilibrium by separation of the phases for 12 h at 25 °C. After phase separation, both the top (IL-rich solution) and bottom (K_3_PO_4_-rich solution) phases were carefully collected. The compositions of the top and bottom phases were determined using the gravimetric method described by Merchuk et al. [58], and the concentrations of the IL components were confirmed using ^1^H-NMR. Each TL was determined using the lever rule to calculate the relationship between the top mass phase composition and the overall system composition. The tie-line length (TLL) was calculated using Equation (1):

(1) $$\mathrm{TLL}=\sqrt{{\left(X_{T}-X_{B}{)}^{2}+(Y_{T}-Y_{B}\right)}^{2}}$$

where X and Y are the weight fraction of the IL and salt, respectively; and the subscript T and B indicate the top and bottom phases, respectively. The TLL of the studied systems increased at alkali conditions (Table S3 in the Supplementary Materials).

### 3.4. NMR Analysis

Mixtures in the biphasic regions with mass fractions of 10% neutral and 15% alkaline phosphate salt, and with 35, 40, and 45% ILs, were prepared. Once phase separation was achieved (as described above), the top and bottom phases were separated. The IL aqueous solutions were prepared with concentrations in the range of 10–60 wt%. Pure ILs, IL solutions, and the top and bottom phases collected from the ABS were loaded solventless in a flame-sealed capillary, and the ^1^H-NMR spectra were collected at 25 °C using *d*_6_-DMSO (dimethyl sulfoxide) as the external lock. Three sets of ^1^H-NMR determinations were performed and the chemical shift deviations of the protons of the ILs, as a function of water and IL concentrations, were determined. The chemical shift deviations of the different mixtures were calculated using Equations (2)–(4):

Δδ_1_ = δ(IL solution) − δ(pure IL) (2)

Δδ_2_ = δ(ABS)_IL_ − δ(pure IL) (3)

Δδ_1_ = δ(ABS)_K3PO4_ − δ(pure IL) (4)

where the subscripts K_3_PO_4_ and IL denote the K_3_PO_4_ (bottom) or IL (top) phase in the ABS.

### 3.5. Extraction and Determination of the Target Herbicides Using the ABS Formed by ILs and pH 7.5 Phosphate Solutions

First, in 10.0 mL centrifuge tubes, known masses of 80 wt% IL, 24 wt% pH 7.5 phosphate solution (prepared as described in the section above), water, and the solution containing a mixture of the three target herbicides (spiked at 4.0 µg/g), were sequentially added to form the ABS with different mass fractions of IL and K_3_PO_4_. The mixtures were stirred for 2 min by vortex at room temperature and then centrifuged at 4500 revolutions per minute (rpm) for 10 min (Sorvall ST8, Thermo Scientific, Waltham, MA, USA). After centrifugation, the centrifuge tube was left on the bench at room temperature for 2 h to form the ABS. The top phase was primarily comprised of IL, the analytes, and water, while the bottom phase was mainly comprised of phosphate salts and water. A blank sample was also prepared and treated according to the procedure mentioned above without adding the target herbicide solutions. The top and bottom phases were carefully separated using plastic syringes, and the volumes and masses of the top and bottom phases were recorded.

The top phases were filtered with a 0.45 µm syringe filter membrane (VWR International, Ville Mont-Royal, QC, Canada) and injected into an HPLC for the separation and quantification of the herbicides. An Agilent 1260 series HPLC system with a quaternary pump, autosampler, and multiwavelength UV-Vis detector (Agilent Technologies, Santa Clara, CA, USA) was used for the determination of the studied herbicides. HPLC separations were performed using an XDB-C_18_ column (Zorbax Eclipse 250 mm × 4.6 mm, 5 µm, Agilent Technologies, Santa Clara, CA, USA) at 25 °C. Gradient elution was performed with water and acetonitrile (ACN) as the mobile phase for the separation of analytes. The analysis started with 30% (*v*/*v*) ACN to 50% in 20 min. The column was then washed by increasing the proportion of ACN from 50% to 85% in 5 min and then to 95% in 1 min, held at that composition for 4 min, and then returning to 30% ACN, followed by a re-equilibration time of 5 min. The flow rate and the injection volume were 1.0 mL/min and 20 μL, respectively. The detection wavelength was 220 nm.

Recovery (R) of the target compounds was used to evaluate the extraction efficiency of the analytes by the ABS and was determined using Equation (5):

(5) $$R\%=\frac{C_{IL}V_{IL}}{C_{0}V_{0}}\times 100\%$$

where *C_IL_* and *C*_0_ are the concentration of the analytes in the IL (top) phase and the initial concentration of the analytes in the spiked sample solution, respectively; and *V_IL_* and *V*_0_ are the volumes of the IL phase and the spiked sample solution, respectively.

## 4. Conclusions

In this manuscript, we demonstrated the ability of neutral and strong alkaline K_3_PO_4_ solutions to salt out choline alkanoate ILs to form ABS. The ability to form ABS with alkaline K_3_PO_4_ solutions decreased in the order [Cho][OAc] ≈ [Cho][Pro] > [Cho][But] > [Cho][Hex], while with neutral K_3_PO_4_ solution [Cho][OAc] would not form an ABS, and the other three ILs performed similarly. All biphasic regions of the ABS decreased with increases in pH. ^1^H-NMR confirmed an anion exchange between the phases, especially at neutral pH. Further studies are needed to fully understand the factors driving ABS formation using [Cho]-based ILs and inorganic salts at neutral pH, including a complete understanding of the speciation occurring in each phase and the probability of anion exchange with the IL anion.

The ABS at neutral pH were then evaluated for herbicide extraction efficiency, due to the instability of the analytes in acidic or basic solutions. A higher affinity of the analytes (i.e., simazine, cyanazine, and atrazine) to the IL-rich phase was observed, with recoveries higher than 60% in all cases. Overall, the ABS formed with [Cho][Pro] and neutral K_3_PO_4_ solution exhibited the highest extraction recovery of the triazine herbicides, with recoveries higher than 90%.

## Figures and Tables

**Table 1:** Aqueous biphasic systems (ABS) formation by choline alkanoate ionic liquids (ILs) and different phosphate solutions ^1^.

	[Cho][OAc]	[Cho][Pro]	[Cho][But]	[Cho][Hex]	[Cho][Oct]
K_3_PO_4_ (pH 14.5)	✓	✓	✓	✓	×
K_3_PO_4_ (pH 7.2)	×	✓	✓	✓	×
Na_2_HPO_4_ (pH 9.0)	×	×	×	×	×
NaH_2_PO_4_ (pH 3.3)	×	×	×	×	×
NaH_2_PO_4_/Na_2_HPO_4_ (pH 5.5)	×	×	×	×	×
NaH_2_PO_4_/Na_2_HPO_4_ (pH 7.0)	×	×	×	×	×

^1^ “✓”: can form ABS. “×”: cannot form ABS.

**Table 2:** Extraction efficiency using the ABS composed of choline alkanoate IL + K_3_PO_4_ + H_2_O at the same concentration of salt (pH 7.5).

ABS IL/K_3_PO_4_ (pH 7.5)	IL (wt%)	K_3_PO_4_ (wt%)	Recovery ± SD ^a^ (%) ^b^		
			Simazine	Cyanazine	Atrazine
[Cho][Pro]/K_3_PO_4_	35.37	9.92	88 (5)	92 (5)	86 (5)
	39.60	10.04	93 (4)	92 (4)	97 (4)
	43.98	9.85	96 (4)	92 (4)	98 (4)
[Cho][But]/K_3_PO_4_	34.35	10.11	52 (5)	54 (5)	58 (5)
	39.70	10.16	69 (6)	68 (5)	77 (5)
	44.82	9.98	68 (6)	62 (5)	75 (6)
[Cho][Hex]/K_3_PO_4_	35.23	9.94	60 (6)	67 (6)	71 (4)
	39.99	10.18	66 (5)	73 (5)	83 (5)
	44.58	10.09	75 (4)	80 (5)	93 (4)

^a^ SD: Standard Deviation; ^b^ Spiked concentration: 4.0 µg/g.

**Table 3:** Chemical properties of the ILs used in this work.

IL						
	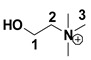 [Cho]^+^	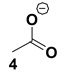 [OAc]^−^	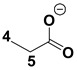 [Pro]^−^	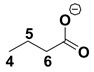 [But]^−^	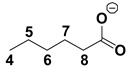 [Hex]^−^	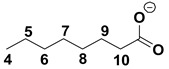 [Oct]^−^
	
Molecular weight (g/mol)		163.2	177.2	191.2	219.2	247.4
Water content (ppm)		4840 ± 125	4960 ± 135	5041 ± 185	5117 ± 168	5132 ± 150

## Data Availability

Data is contained within the article or supplementary material. Additional data available on request to the authors.

## Supplementary Materials

Ionic liquids characterization (^1^H-NMR, ^13^C-NMR, and FTIR) (SI, Section 1, Figure S1); Binodal data of ABS (SI, Section 2, Figures S2–S4, Tables S1–S3); ^1^H-NMR spectra of the two phases in the ABS (SI, Section 3, Figures S5–S10, Table S4); Extraction and determination of herbicides by HPLC (SI, Section 4, Figures S11–S13, Table S5). Reference [59] is cited in the Supplementary Materials.

## Sample Availability

Samples of the compounds are not available from the authors.
