# Supplementary material for: Phase Behavior of Aqueous Biphasic Systems with Choline Alkanoate Ionic Liquids and Phosphate Solutions: The Influence of pH
Source: Molecules. 2021 Mar 18;26(6):1702. doi: 10.3390/molecules26061702 (PMC8003127; doi:10.3390/molecules26061702)
Supplement: Supplementary file 1 [file molecules-26-01702-s001.zip › molecules-1122151-supplementary.pdf]

Supplementary Information

Phase Behavior of Aqueous Biphasic Systems with Choline Alkanoate Ionic Liquids and Phosphate Solutions: The Influence of pH

Paula Berton,[a,b] Hongzhe Tian,*[a,c] Robin D. Rogers*[a,d]

[a] *Department of Chemistry, McGill University, 801 Sherbrooke St. West Montreal, QC Canada H3A 0B8*

[b] *Chemical and Petroleum Engineering Department, University of Calgary, 2500 University Drive NW, Calgary, Alberta T2N 1N4, Canada*

[c] *Plant Protection College, Shenyang Agricultural University, 120 Dongling Road, Shenyang 110161, P.R. China*

[d] *525 Solutions Inc., P.O. Box 2206, Tuscaloosa, AL USA 35403*

Email: tiansyau@163.com; Robin.Rogers@525Solutions.com

1. Ionic Liquids Characterization: ^{1}H-NMR, ^{13}C-NMR and FTIR Data

[Cho][OAc]: ^{1}H-NMR (500 MHz, [d$_6$] DMSO, TMS): δ= 1.62 (s, 3H, –OOCCH$_3$), 3.13 (s, 9H, –NCH$_3$), 3.43 (t, 2H, –NCH$_2$), 3.84 (d, 2H, –OCH$_2$) ppm. ^{13}C-NMR (100 MHz, [d$_6$] DMSO), δ= 25.5, 53.5, 55.4, 67.7, 173.8 ppm.

[Cho][Pro]: ^{1}H-NMR (500 MHz, [d$_6$] DMSO): δ = 0.88 (t, 3H, –CH$_3$), 1.81 (m, 2H, –CH$_2$COO), 3.14 (s, 9H, –NCH$_3$), 3.44 (m, 2H, –NCH$_2$), 3.85 (m, 2H, –OCH$_2$) ppm. ^{13}C-NMR (100 MHz, [d$_6$] DMSO): δ = 11.91 (–CH$_3$), 31.84(–CH$_2$COO), 53.54 (–NCH$_3$), 55.34 (–NCH$_2$), 67.85 (–OCH$_2$), 176.93 (-COO$^-$) ppm.

[Cho][But]: ^{1}H-NMR (500 MHz, D$_2$O): δ = 0.80 (t, 3H, –CH$_3$), 1.46 (m, 2H, –CH$_2$CH$_2$COO), 2.06 (t, 2H, –CH$_2$COO), 3.11 (s, 9H, –NCH$_3$), 3.43 (m, 2H, –NCH$_2$), 3.97 (m, 2H, –OCH$_2$) ppm; ^{13}C-NMR (100 MHz, D$_2$O): δ = 13.22 (–CH$_3$), 19.34 (–CH$_2$CH$_2$COO), 39.58 (–CH$_2$COO), 53.83 (–NCH$_3$), 55.56 (–NCH$_2$), 67.38 (–OCH$_2$), 184.01 (–COO$^-$) ppm.

[Cho][Hex]: ^{1}H-NMR (500 MHz, [d$_6$] DMSO): δ = 0.84 (t, 3H, –CH$_3$CH$_2$), 1.18 (m, 4H, –CH$_3$(CH$_2$)$_2$–), 1.40 (m, 2H, –CH$_2$CH$_2$COO), 1.84 (t, 2H, –CH$_2$COO), 3.14 (s, 9H, –N(CH$_3$)$_3$), 3.43 (m, 2H, –NCH$_2$), 3.84 (m, 2H, –OCH$_2$) ppm. ^{13}C-NMR (100 MHz, [d$_6$] DMSO): δ = 14.49 (–CH$_3$CH$_2$–), 22.66 (–CH$_3$CH$_2$–), 26.68 (–CH$_3$CH$_2$CH$_2$–), 32.21 (–CH$_2$CH$_2$COO), 38.97 (–CH$_2$COO), 53.51 (–N(CH$_3$)$_3$), 55.34 (–NCH$_2$), 67.84 (–OCH$_2$), 176.43 (–COO$^-$) ppm.

[Cho][Oct]: ^{1}H-NMR (500 MHz, [d$_6$] DMSO): δ = 0.85 (t, 3H, –CH$_3$CH$_2$), 1.21 (m, 8H, –CH$_3$(CH$_2$)$_4$–), 1.39 (m, 2H, –CH$_2$CH$_2$COO), 1.83 (t, 2H, –CH$_2$COO), 3.13 (s, 9H, –N(CH$_3$)$_3$), 3.43 (m, 2H, –NCH$_2$), 3.84(m, 2H, –OCH$_2$) ppm. ^{13}C-NMR (100 MHz, [d$_6$] DMSO): δ = 14.44 (–CH$_3$CH$_2$–), 22.60 (–CH$_3$CH$_2$–), 27.02 (–CH$_3$CH$_2$CH$_2$–), 29.30 (–CH$_3$CH$_2$ CH$_2$CH$_2$–), 29.91 (–CH$_3$CH$_2$ CH$_2$ CH$_2$CH$_2$–), 31.85 (–CH$_2$CH$_2$COO), 39.01 (–CH$_2$COO), 53.54 (–N(CH$_3$)$_3$), 55.34 (–NCH$_2$), 67.84 (–OCH$_2$), 176.27 (–COO$^-$) ppm.

The FTIR spectra matched the reported spectra [1]; specific peaks at 3145, 3025, 2850, 1573, 1386, and 1080 cm^{-1}.

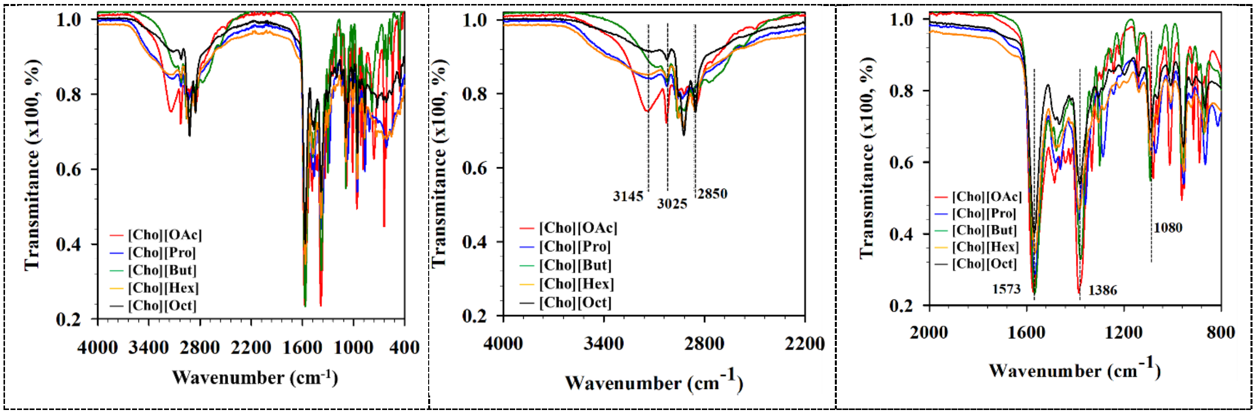

Figure S1. FTIR spectra of the synthesized ILs.

2. Binodal Data of the ABS

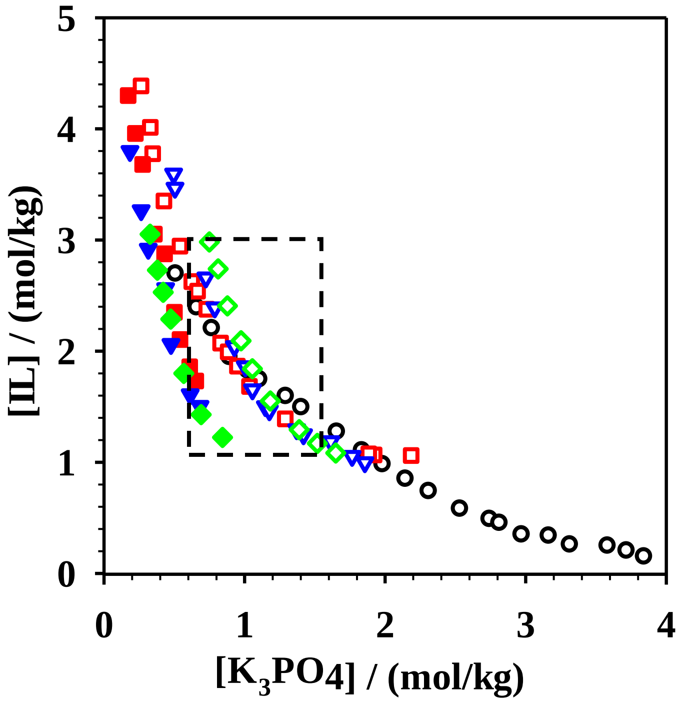

Figure S2. Phase diagrams of the ABS formed by of [Cho][OAc] (●), [Cho][Pro] (■), [Cho][But] (▼), and [Cho][Hex] (◆) and K_3PO_4 at pH 7.2 (filled symbols) and pH 14.5 (open symbols).

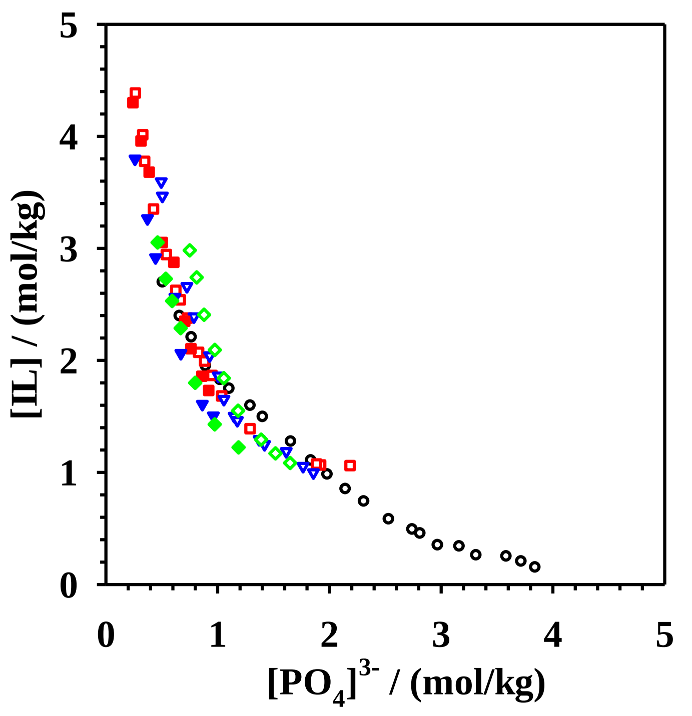

Figure S3. Phase diagrams of the ABS formed by of [Cho][OAc] (●), [Cho][Pro] (■), [Cho][But] (▼), and [Cho][Hex] (◆) and K_3PO_4 at pH 7.2 (filled symbols) and pH 14.5 (open symbols) based on phosphate concentration as horizontal axis.

Table S1. Experimental weight fraction data for the system composed of neutral K_3PO_4 solution (1) + IL (2) + H_2O (3) at room temperature and atmospheric pressure.

[Cho][Pro]		[Cho][But]		[Cho][Hex]	
**100w_1**	**100w_2**	**100w_1**	**100w_2**	**100w_1**	**100w_2**
2.0883	76.2076	2.2401	72.6343	3.9813	66.9091
2.7043	70.1546	3.2040	62.4507	4.6121	59.8201
3.3354	65.2198	3.8240	55.7957	5.1062	55.4638
4.3481	54.0968	5.3172	49.0199	5.7613	50.1533
5.2342	50.9602	5.7784	39.4444	6.8815	39.4638
6.0724	41.6331	7.4334	30.7692	8.3861	31.3192
6.5573	37.3039	8.2826	28.7741	10.2244	26.8368
7.3999	32.9482				
7.9181	30.6824				

Table S2. Experimental weight fraction data for the system composed of alkaline K_3PO_4 (1) + IL (2) + H_2O (3) at room temperature and atmospheric pressure.

[Cho][Pro]		[Cho][But]		[Cho][Hex]	
$100w_1$	$100w_2$	$100w_1$	$100w_2$	$100w_1$	$100w_2$
3.2005	77.7450	6.0076	68.7464	9.0981	65.376
3.9957	71.1263	6.1288	66.2986	9.8517	60.0782
4.2052	66.9297	6.9568	60.5348	10.6495	52.7845
5.1689	59.3903	7.4905	55.3247	11.8195	45.8994
6.5587	52.1940	8.4917	50.8652	12.7974	40.3782
7.5800	46.5332	9.5579	45.7103	14.3511	33.9995
8.0801	45.0185	11.2513	39.0572	16.8459	28.3509
8.8691	42.1376	12.1954	35.5941	18.4058	25.644
10.0575	36.7282	12.8121	31.6222	19.9952	23.7684
10.7105	35.3304	13.9227	28.7264		
11.5131	33.0638	14.2644	28.0112		
12.5582	29.8323	16.6263	24.767		
15.6406	24.6471	17.2161	23.9039		
22.8486	19.0623	19.5687	22.6959		
23.3101	18.9010	21.4011	20.1819		
26.5005	18.8055	22.5126	19.0826		

Table S3. Compositions of the ABSs used for NMR analysis.

ABS	Overall (wt%)		Top phase (wt%)			Bottom phase (wt%)			100 TLL
	IL	K_3PO_4	IL	K_3PO_4	pH	IL	K_3PO_4	pH	
[Ch][Pro]/K_3PO_4 (pH 7.2)	35.00	10.36	43.01	5.70	8.47	18.40	19.01	8.20	27.98
	39.92	9.94	50.02	4.60	8.42	16.95	21.35	8.16	37.07
	45.20	10.15	60.30	3.35	8.45	14.50	25.80	8.18	51.01
[Ch][But]/K_3PO_4 (pH 7.2)	35.16	9.96	74.25	2.75	8.42	23.12	13.20	8.13	52.18
	40.38	10.09	75.97	2.54	8.43	22.95	13.46	8.15	54.14
	45.07	9.95	78.51	2.32	8.40	21.32	14.05	8.12	58.38
[Ch][Pro]/K_3PO_4 (pH 14.5)	35.18	14.97	56.20	5.51	13.50	20.22	21.50	14.31	39.37
	39.80	16.04	63.10	4.85	13.55	19.05	24.80	14.42	48.36
	45.21	15.07	69.40	4.01	13.52	18.01	28.10	14.35	56.76
[Ch][But]/K_3PO_4 (pH 14.5)	34.97	15.47	65.25	6.53	13.80	22.02	20.20	14.22	45.33
	40.00	15.53	67.98	6.34	13.81	21.75	20.66	14.24	48.39
	44.46	15.17	70.51	6.02	13.78	21.52	21.55	14.20	51.39

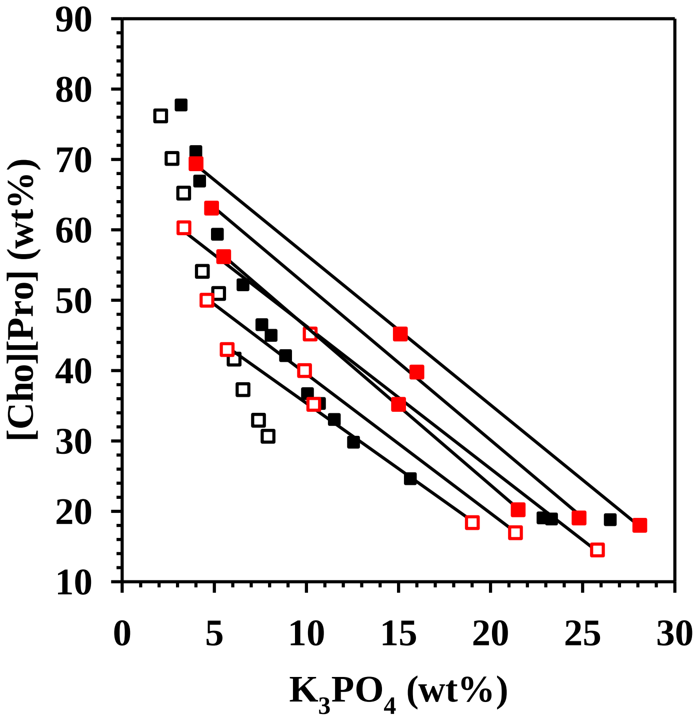

Figure S4. Binodal curves and tie-line data for [Cho][Pro]/K_3PO_4 ABS at pH 7.2 (open symbols) and 14.5 (filled symbols) with the overall compositions used for the NMR study (red symbols).

3. ^{1}H-NMR spectra of the two phases in the ABS

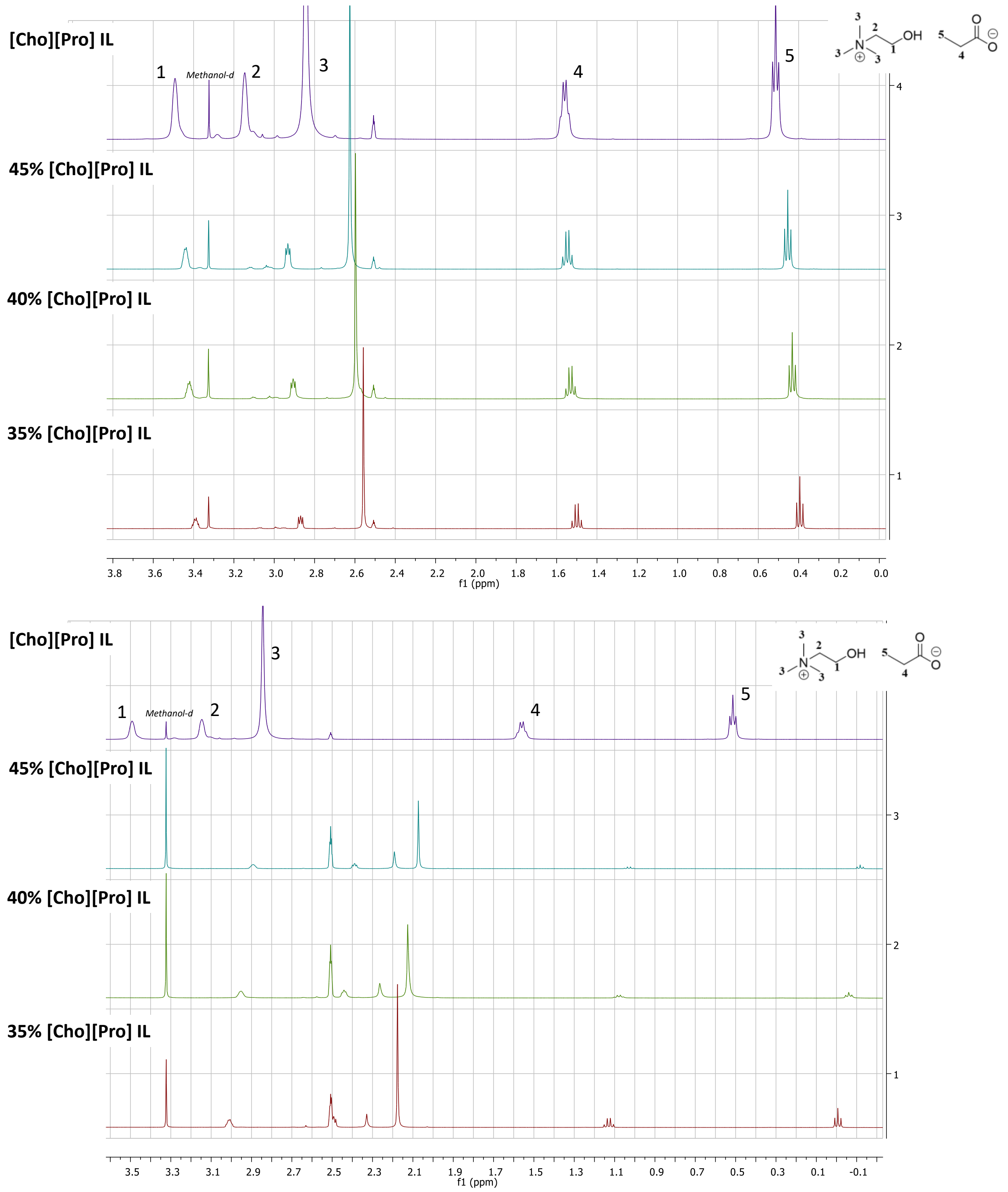

Figure S5. ^{1}H-NMR spectra of the top (*top*) and bottom (*bottom*) phases of ABS formed with [Cho][Pro] and 10 wt% K_3PO_4 (pH 7.2), compared with pure [Cho][Pro] IL.

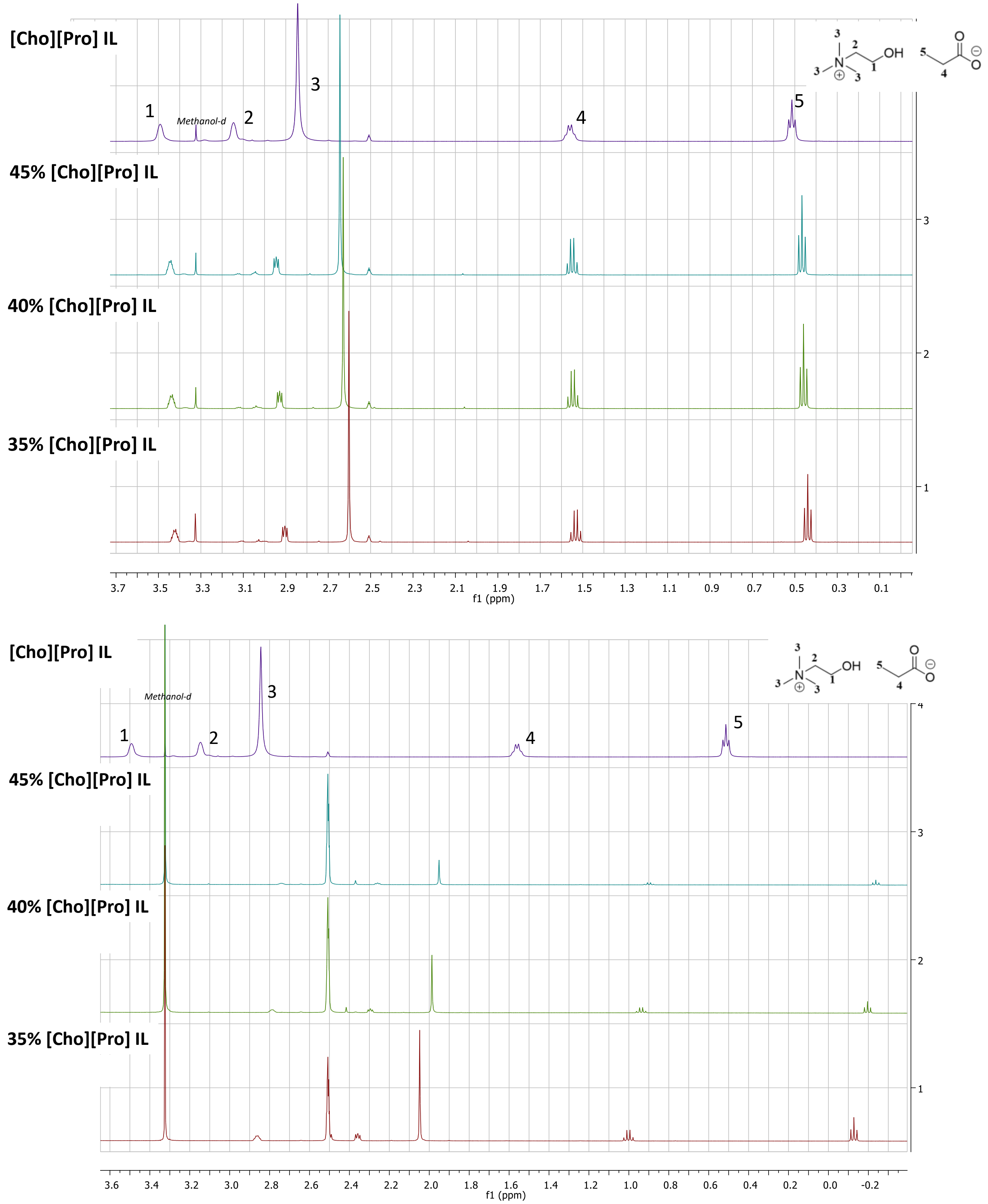

Figure S6. ^{1}H-NMR spectra of the top (*top*) and bottom (*bottom*) phases of ABS formed with [Cho][Pro] and 15 wt% K_3PO_4 (pH 14.5), compared with pure [Cho][Pro] IL.

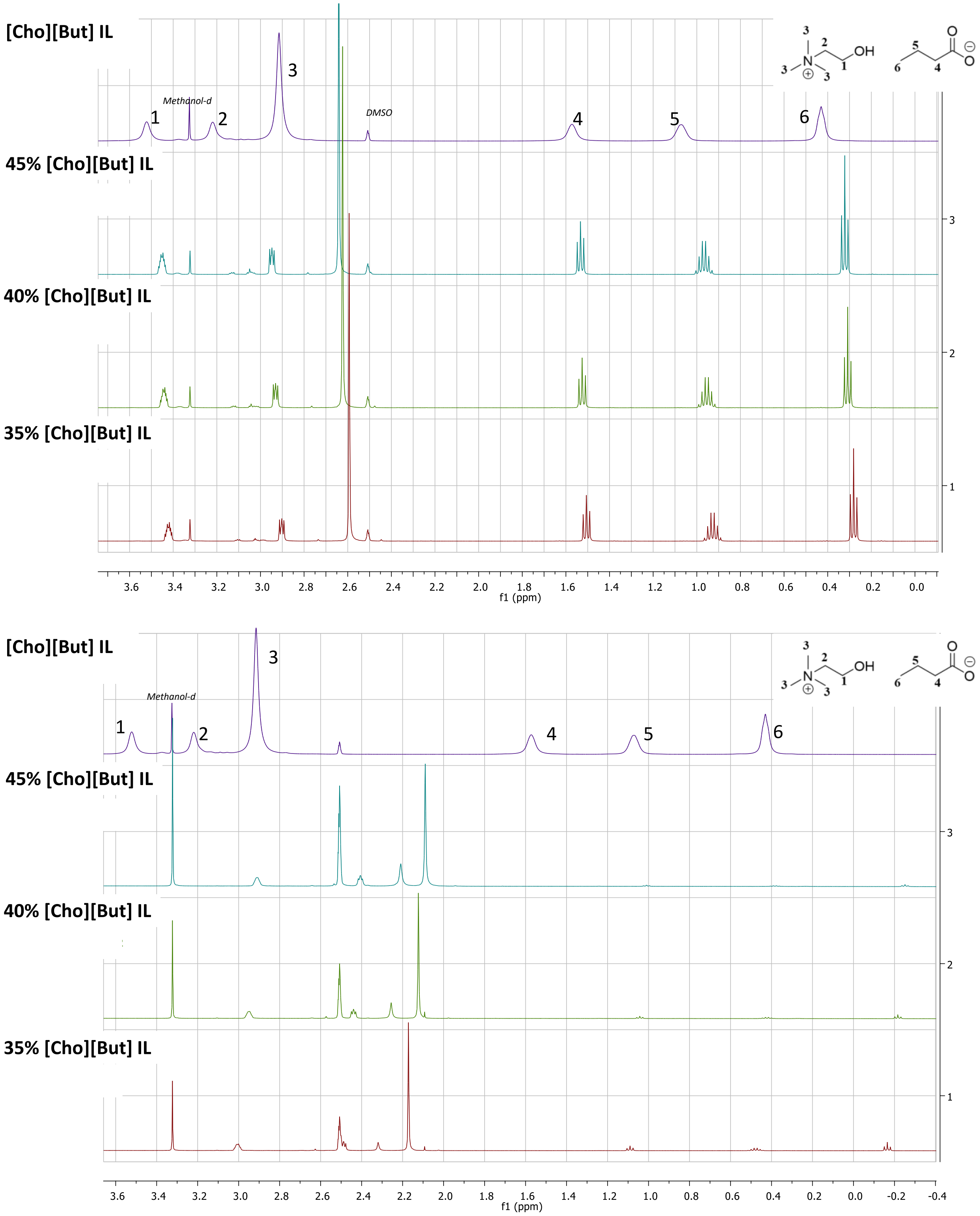

Figure S7. ^{1}H-NMR spectra of the top (*top*) and bottom (*bottom*) phases of ABS formed with [Cho][But] and 10 wt% K_3PO_4 (pH 7.2), compared with pure [Cho][But] IL.

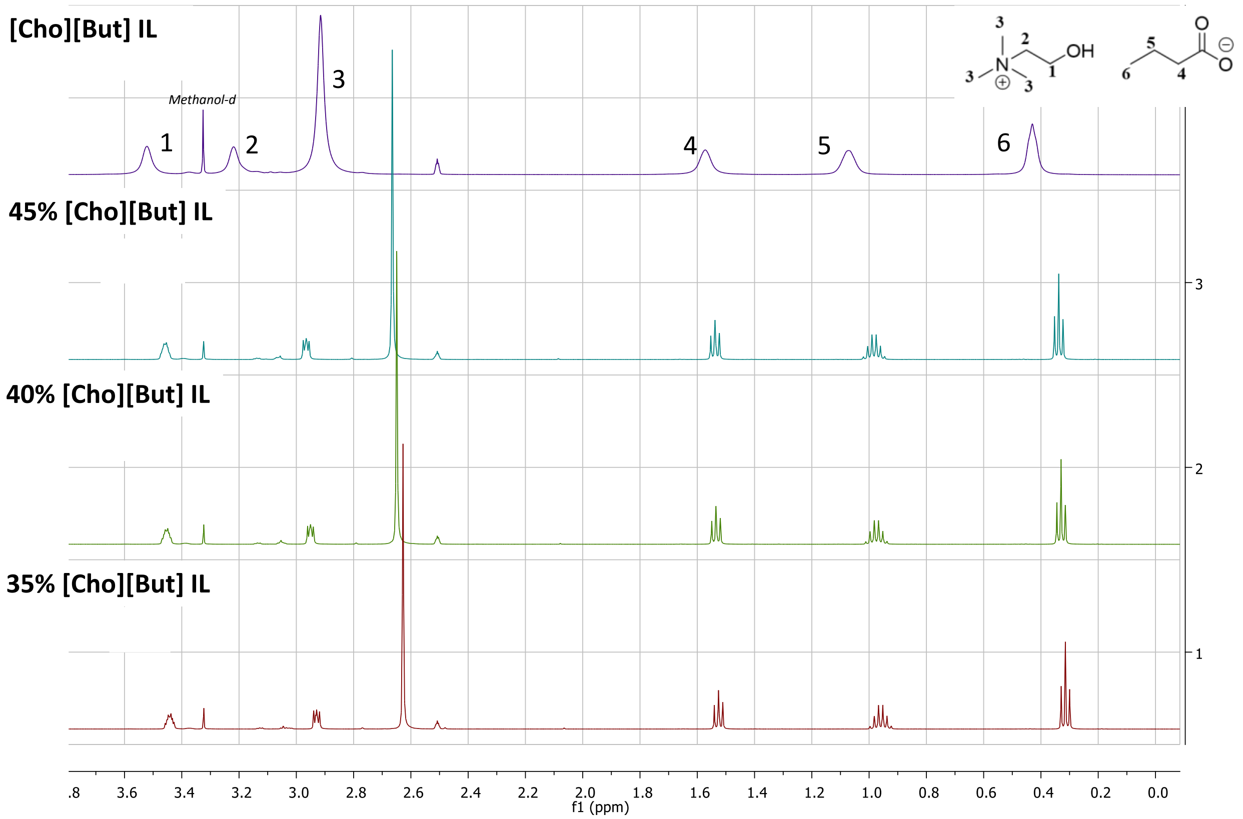

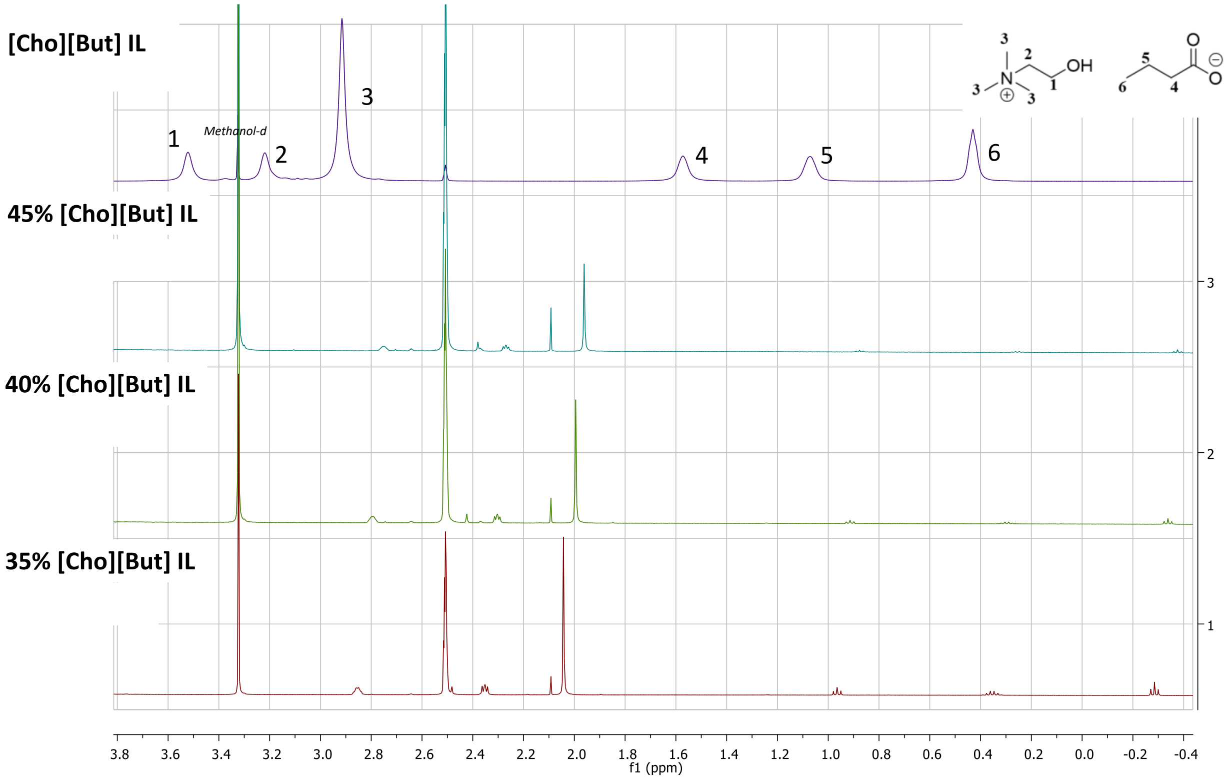

Figure S8. ^{1}H-NMR spectra of the top (*top*) and bottom (*bottom*) phases of ABS formed with [Cho][But] and 15 wt% K_3PO_4 (pH 14.5), compared with pure [Cho][But] IL.

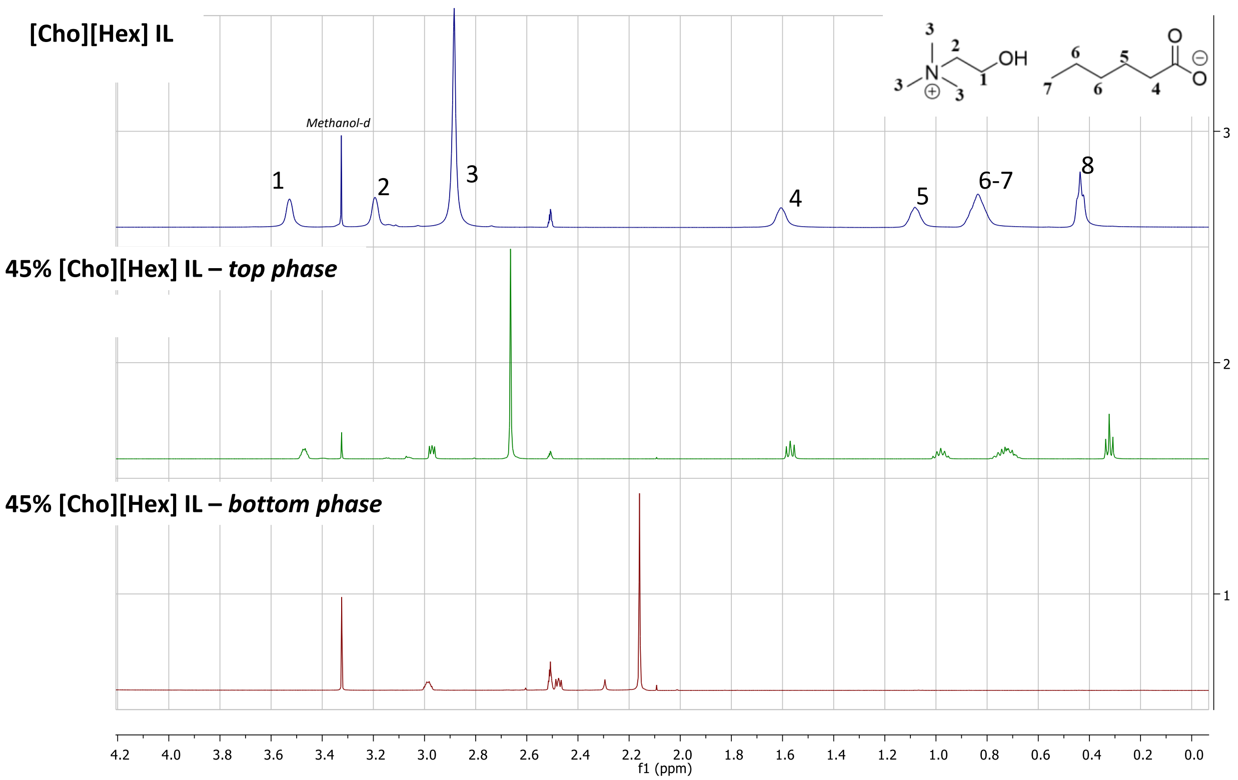

Figure S9. ^{1}H-NMR spectra of the top (*top*) and bottom (*bottom*) phases of ABS formed with [Cho][Hex] and 10 wt% K_3PO_4 (pH 7.2), compared with pure [Cho][Hex] IL.

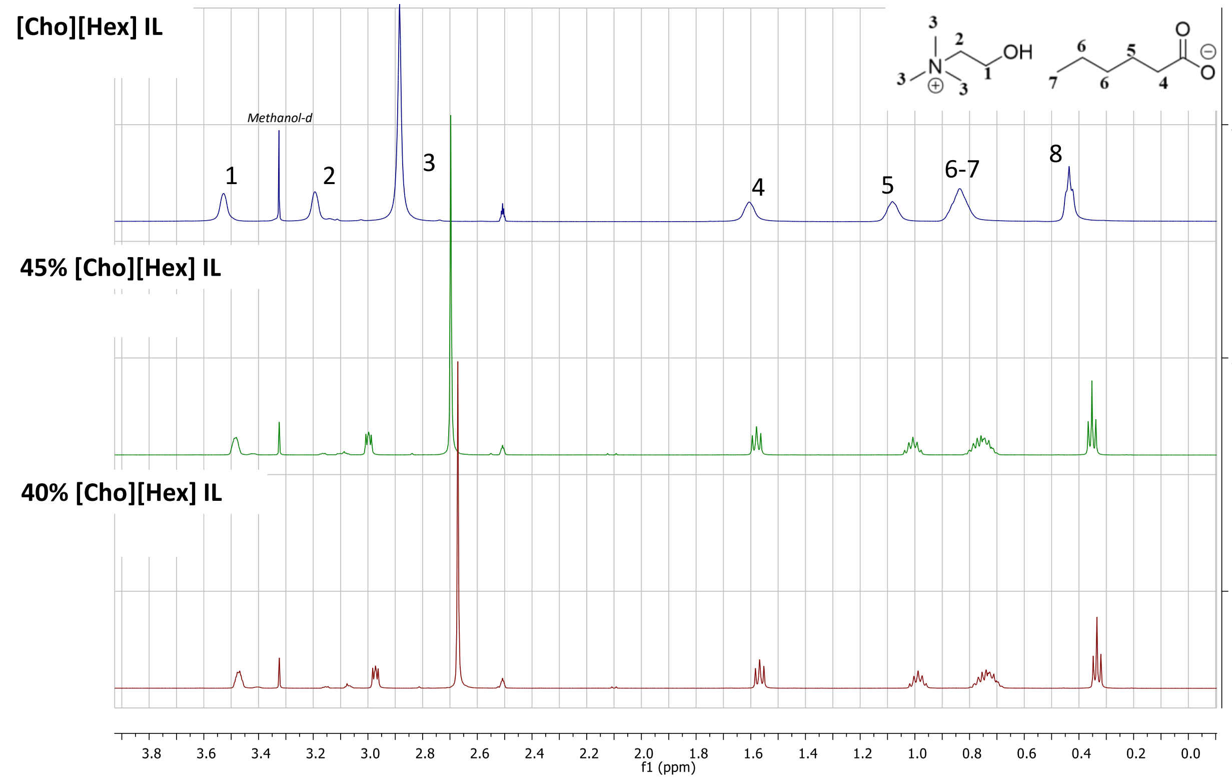

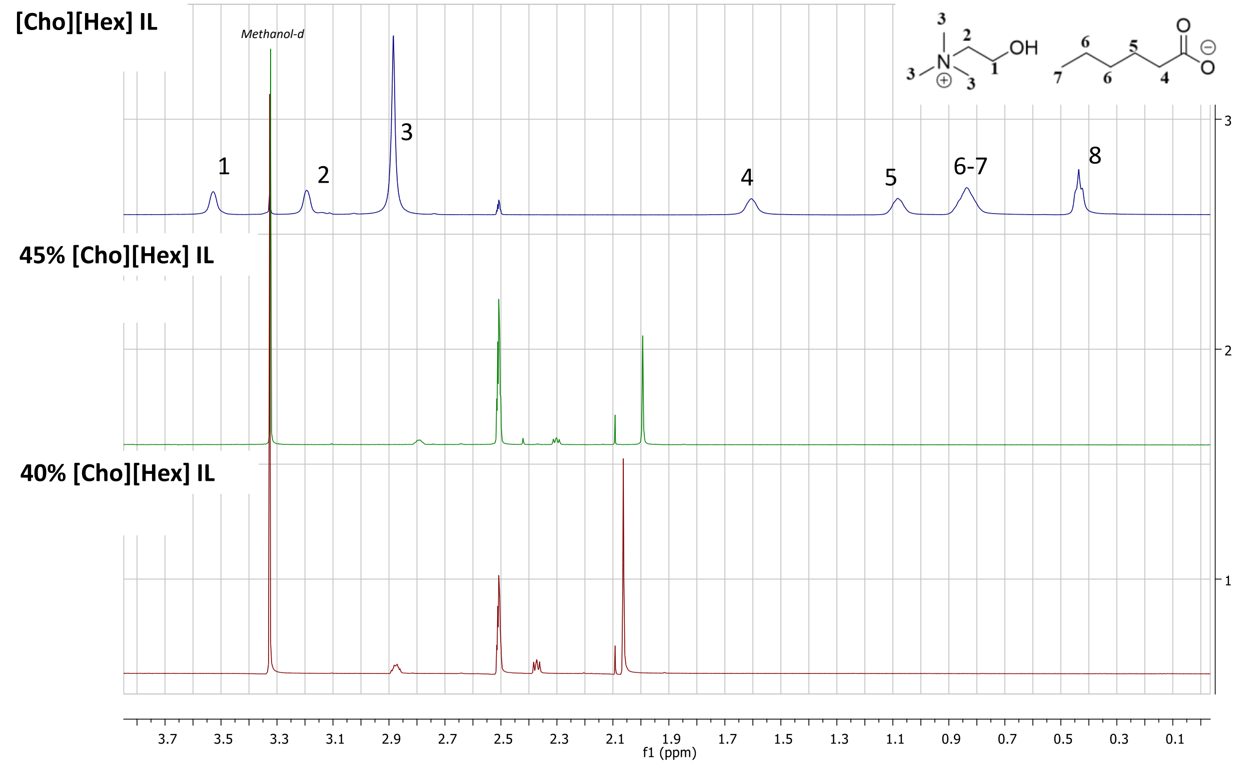

Figure S10. ^{1}H-NMR spectra of the top (*top*) and bottom (*bottom*) phases of ABS formed with [Cho][Hex] and 15 wt% K_3PO_4 (pH 14.5), compared with pure [Cho][Hex] IL.

Table S4. Comparison of the integral areas of the protons (H1:H4) in the ABS

Initial Composition (wt%)	$C:A_{[H1:H4]}$	
	**Top Phase**	**Bottom Phase**
**Pure [Cho][Pro]**	1:1	
[Cho][Pro] 35.0%/10.4% K_3PO_4 (pH 7.2)	1:1	1:0.5
[Cho][Pro] 39.9%/9.9% K_3PO_4 (pH 7.2)	1:1	1:0.4
[Cho][Pro] 45.2%/10.2% K_3PO_4 (pH 7.2)	1:1	1:0.3
[Cho][Pro] 35.2%/15.0% K_3PO_4 (pH 14.5)	1:1	1:0.9
[Cho][Pro] 39.8%/16.0% K_3PO_4 (pH 14.5)	1:1	1:1
[Cho][Pro] 45.2%/15.1% K_3PO_4 (pH 14.5)	1:1	1:1
**Pure [Cho][But]**	1:1	
[Cho][But] 35.2%/10.0% K_3PO_4 (pH 7.2)	1:1	1:0.3
[Cho][But] 40.4%/10.1% K_3PO_4 (pH 7.2)	1:1	1:0.2
[Cho][But] 45.1%/9.9% K_3PO_4 (pH 7.2)	1:1	1:0.1
[Cho][But] 35.0%/15.5% K_3PO_4 (pH 14.5)	1:1	1:0.4
[Cho][But] 40.0%/15.5% K_3PO_4 (pH 14.5)	1:1	1:0.3
[Cho][But] 44.5%/15.2% K_3PO_4 (pH 14.5)	1:1	1:0.4
**Pure [Cho][Hex]**	1:1	
[Cho][Hex] 44.7%/10.2% K_3PO_4 (pH 7.2)	1:1	nd[a]
[Cho][Hex] 40.1%/14.8% K_3PO_4 (pH 14.5)	1:1	nd
[Cho][Hex] 45.2%/15.4% K_3PO_4 (pH 14.5)	1:1	nd

[a] nd: Anion not detected

4. Extraction and determination of herbicides by HPLC

Table S5. Viscosity of aqueous solutions of the ILs at room temperature

IL Concentration (%)	Viscosity ± SD (cP)
25.92% [Cho][Pro]	1.897 (0.001)
41.72% [Cho][Pro]	3.557 (0.011)
62.09% [Cho][Pro]	11.798 (0.039)
25.19% [Cho][But]	2.148 (0.008)
38.52% [Cho][But]	3.641 (0.007)
57.78% [Cho][But]	10.308 (0.055)
23.70% [Cho][Hex]	2.080 (0.003)
37.62% [Cho][Hex]	3.500 (0.004)
56.42% [Cho][Hex]	11.523 (0.033)

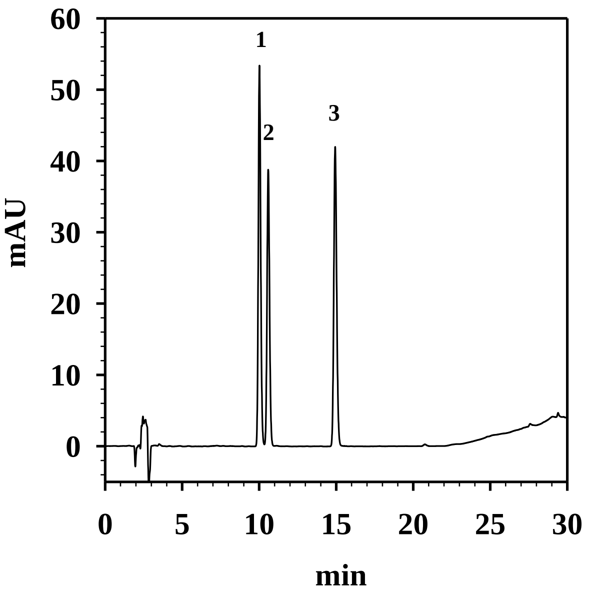

Figure S11. Chromatogram of the pesticide's standard solution (5 μg/mL) separated by HPLC. 1-Simazine; 2-Cyanazine; 3-Atrazine.

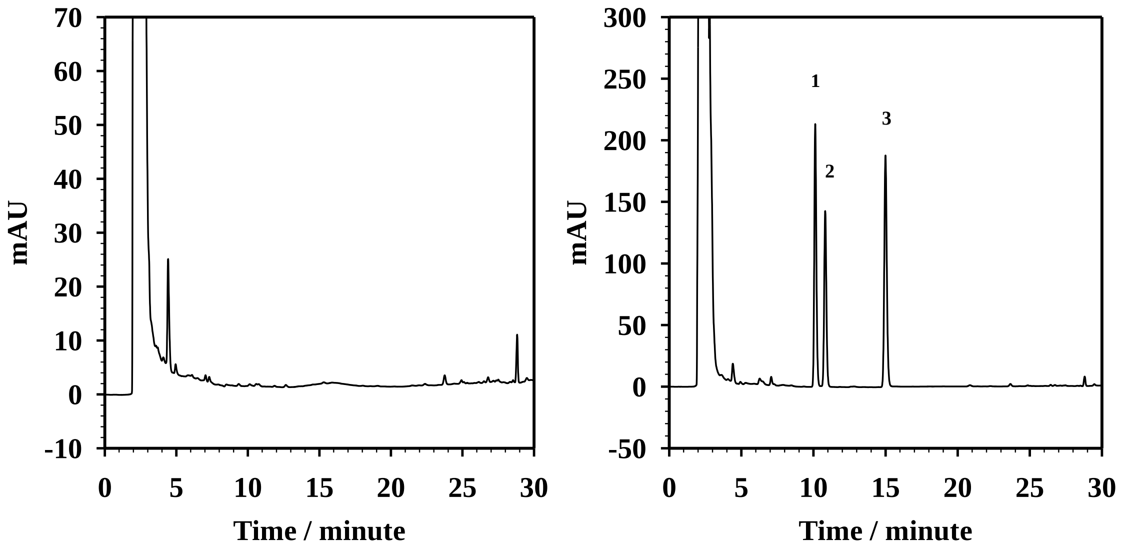

Figure S12. Chromatograms of the ABS (35.0 wt% [Cho][Pro], 10.0 wt % K_3PO_4, pH 7.5) top phases (*Left*) in the original sample, and (*Right*) in the spiked (10.0 µg/g) sample. 1-Simazine; 2-Cyanazine; 3-Atrazine.

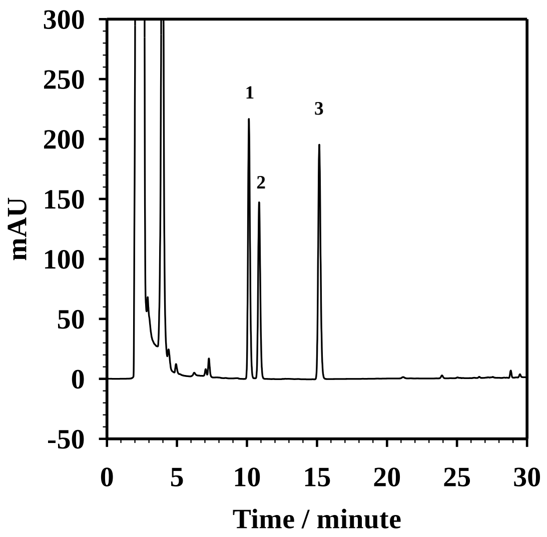

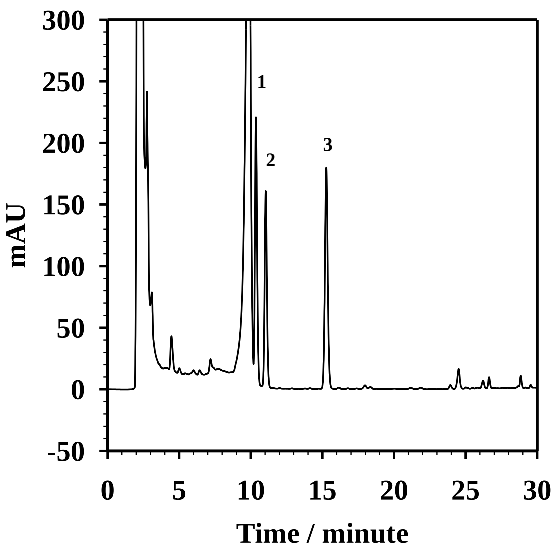

Figure S13. Chromatograms of the top phases (*Left*) in the ABS spiked sample (35.0 wt% [Cho][But], 10.0 wt% K_3PO_4, pH 7.5), and (*Right*) in the ABS spiked sample (35.0 wt% [Cho][Hex], 10.0 wt% K_3PO_4, pH 7.5). Spiked concentration 10.0 µg/g. 1-Simazine; 2-Cyanazine; 3-Atrazine.

Reference

1. Kalla, R. M. N.; Lim, J.; Bae, J.; Kim, I. Sulfated choline ionic liquid-catalyzed acetamide synthesis by grindstone method. *Tetrahedron Lett.* **2017**, *58*, 1595-1599.
